# Bidirectional Human–Swine Transmission of Seasonal Influenza A(H1N1)pdm09 Virus in Pig Herd, France, 2018

**DOI:** 10.3201/eid2510.190068

**Published:** 2019-10

**Authors:** Amélie Chastagner, Vincent Enouf, David Peroz, Séverine Hervé, Pierrick Lucas, Stéphane Quéguiner, Stéphane Gorin, Véronique Beven, Sylvie Behillil, Philippe Leneveu, Emmanuel Garin, Yannick Blanchard, Sylvie van der Werf, Gaëlle Simon

**Affiliations:** French Agency for Food, Environmental and Occupational Health, and Safety, Ploufragan, France (A. Chastagner, S. Hervé, P. Lucas, S. Quéguiner, S. Gorin, V. Beven, Y. Blanchard, G. Simon);; Institut Pasteur, Paris, France (V. Enouf, S. Behillil, S. van der Werf);; Atlantic Vétérinaires, Ancenis, France (D. Peroz);; CEVA Santé Animale SA, Libourne, France (P. Leneveu);; Coop de France, Paris (E. Garin);; Plateforme Epidémiosurveillance Santé Animale, Lyon, France (E. Garin)

**Keywords:** influenza, influenza A(H1N1)pdm09, H1N1pdm09, H1N1, interspecies transmission, pandemic, zoonotic disease, reverse zoonosis, swine, human, zoonoses, transmission, France, respiratory infections, case report, viruses, farms, human–swine transmission, swine–human transmission, biosecurity

## Abstract

In 2018, a veterinarian became sick shortly after swabbing sows exhibiting respiratory syndrome on a farm in France. Epidemiologic data and genetic analyses revealed consecutive human-to-swine and swine-to-human influenza A(H1N1)pdm09 virus transmission, which occurred despite some biosecurity measures. Providing pig industry workers the annual influenza vaccine might reduce transmission risk.

In April 2009, a novel influenza A virus (IAV) emerged in humans in North America and spread in the human population worldwide, leading to the first pandemic of the 21st century (*1*). This virus, influenza A(H1N1)pdm09 (pH1N1), suspected to have resulted from reassortment among IAVs of swine origin, was rapidly transmitted to pig populations. This virus became seasonal in humans (*2*) and enzootic in several pig populations, including those in Europe (*3*). Moreover, phylogenetic analyses suggest de novo human-to-swine pH1N1 transmission occurs during seasonal epidemics (*4*–*6*). In this study, we provide evidence of bidirectional transmission of pH1N1 between humans and pigs in a herd located in France.

## The Study

In January of the 2017–18 seasonal influenza epidemic in humans, a farmer reported to a veterinarian an acute respiratory outbreak in the pregnant sows of his farrow-to-wean herd. The animals of this herd (≈1,000 sows) were not vaccinated against swine IAVs. The sows exhibited an influenza-like illness (ILI) of usual intensity (i.e., hyperthermia, apathy, dyspnea, sneezing, and coughing that did not last for >2–3 days for individual animals). On January 17, the veterinarian and a technician handled the animals and, using nasal swabs (MW950Sent2mL Virocult; Kitvia, https://www.kitvia.com), collected samples from 3 sows (sample nos. 180028-1, 180028-2, 180028-3), as specified by the National Network for Surveillance of Type A Influenza Virus in Swine (*7*). The veterinarian (72 hours later) and technician (48 hours later), both not vaccinated against seasonal influenza, had ILI symptoms (i.e., tiredness, runny nose, chills). Neither reported close contact with humans with ILI before their symptom onset. On days 5 and 6 after handling the pigs, the veterinarian self-collected nasal swab samples (sample nos. 180130-1 and 180130-2).

The veterinarian submitted the nasal swab samples from sows to a local veterinary laboratory to determine the diagnosis. This laboratory used quantitative reverse transcription PCR of the influenza matrix gene for IAV detection (*8*). All 3 samples were positive for IAV and were sent to the National Reference Laboratory (Ploufragan, France) for subtyping. Here, we typed the isolates’ hemagglutinin (HA) and neuraminidase genes by using quantitative reverse transcription PCRs specific to swine IAV lineages known to circulate in the pig populations in France (*8*). The HA and neuraminidase genes we identified were exclusively those of pH1N1. We propagated sample no. 180028-2 through Madin-Darby canine kidney cells (1 passage) to obtain isolate A/sw/France/53-180028/2018. We sequenced the whole genome of this virus using an Ion Proton Sequencer (Thermo Fisher Scientific, https://www.thermofisher.com) at the Next-Generation Sequencing Platform of the French Agency for Food, Environmental and Occupational Health, and Safety (Ploufragan, France) (Appendix).

In parallel, we amplified the 8 virus genome segments from the 2 veterinarian self-collected nasal swabs and the 3 sow samples using universal primers (*9*) at the National Reference Center for Respiratory Viruses (Paris, France). Then, we sequenced them on a NextSeq 500 System (Illumina Inc., https://www.illumina.com) at the Mutualized Platform for Microbiology (Paris, France) (Appendix). After cleaning reads, we excluded from analysis data from sample no. 180028-1 because of its low number of residual reads.

In all, we obtained 5 sets of 8 genomic segment sequences (BioProject no. PRJNA507096): 1 from A/sw/France/53-180028/2018, 2 from pigs (sample nos. 180028-2 and 180028-3), and 2 from a human (sample nos. 180130-1 and 180130-2). We found only 3 nucleotide ambiguities supported by >30% of reads: an A-G (45.3%) mixture at position 1,667 in the polymerase basic 2 gene of sample no. 180028-2 and a T-C (49.3%) mixture at position 862 and C-A (34.9%) mixture at position 867 in the HA gene of sample no. 180130-1 (nucleotide numbering starting from first position of coding sequence for all). Excluding these ambiguities, the 5 virus genomes were 100% identical, regardless of source or sequencing pipeline. We compared these virus sequences with those of other pH1N1 strains available in the GISAID database (https://www.gisaid.org) using the integrated BLAST program; the highest similarities (up to 99.94% identity) were found with a pH1N1 isolate identified in a population in France during the 2017–18 winter influenza season (Table). We performed maximum-likelihood phylogenetic analyses that included pH1N1 viruses isolated from pigs and humans in France during 2009–2018. Whatever the genomic segment used, be that encoding HA (Figure) or others (data not shown), the isolates from our case study were more closely related to seasonal influenza isolates than isolates from the swine-specific lineage identified in France during 2015–2016 (*6*), confirming our BLAST results.

**Table:** Influenza A(H1N1)pdm09 strains closest related to A/sw/France/53-180028/2018 isolated from a sow in France, 2018*

Influenza A(H1N1)pdm09 strain†	Collection date	Percentage identity								
		Segment no. (gene)								Whole genome
		1 (PB2)	2 (PB1)	3 (PA)	4 (HA)	5 (NP)	6 (NA)	7 (M)	8 (NS)	
A/Haute Normandie/1985/2017	2017 Dec 28	99.96	99.91	100.00	99.88	100.00	99.93	100.00	99.77	99.94
A/Dijon/181/2018	2017 Dec 31	99.96	99.91	99.95	99.82	100.00	99.86	100.00	99.88	99.94
A/Alsace/560/2018	2018 Jan 22	99.91	99.96	100.00	99.82	100.00	99.93	100.00	99.77	99.93
A/Paris/1767/2017	2017 Dec 15	99.96	99.96	99.95	99.76	100.00	100.00	99.90	99.77	99.92

*HA, hemagglutinin; M, matrix; NA, neuraminidase; NP, nucleoprotein; NS, nonstructural protein; PA, polymerase acidic; PB1, polymerase basic 1; PB2, polymerase basic 2. †Sequences available in GISAID database (https://www.gisaid.org) and selected using integrated BLAST program.

**Figure:**
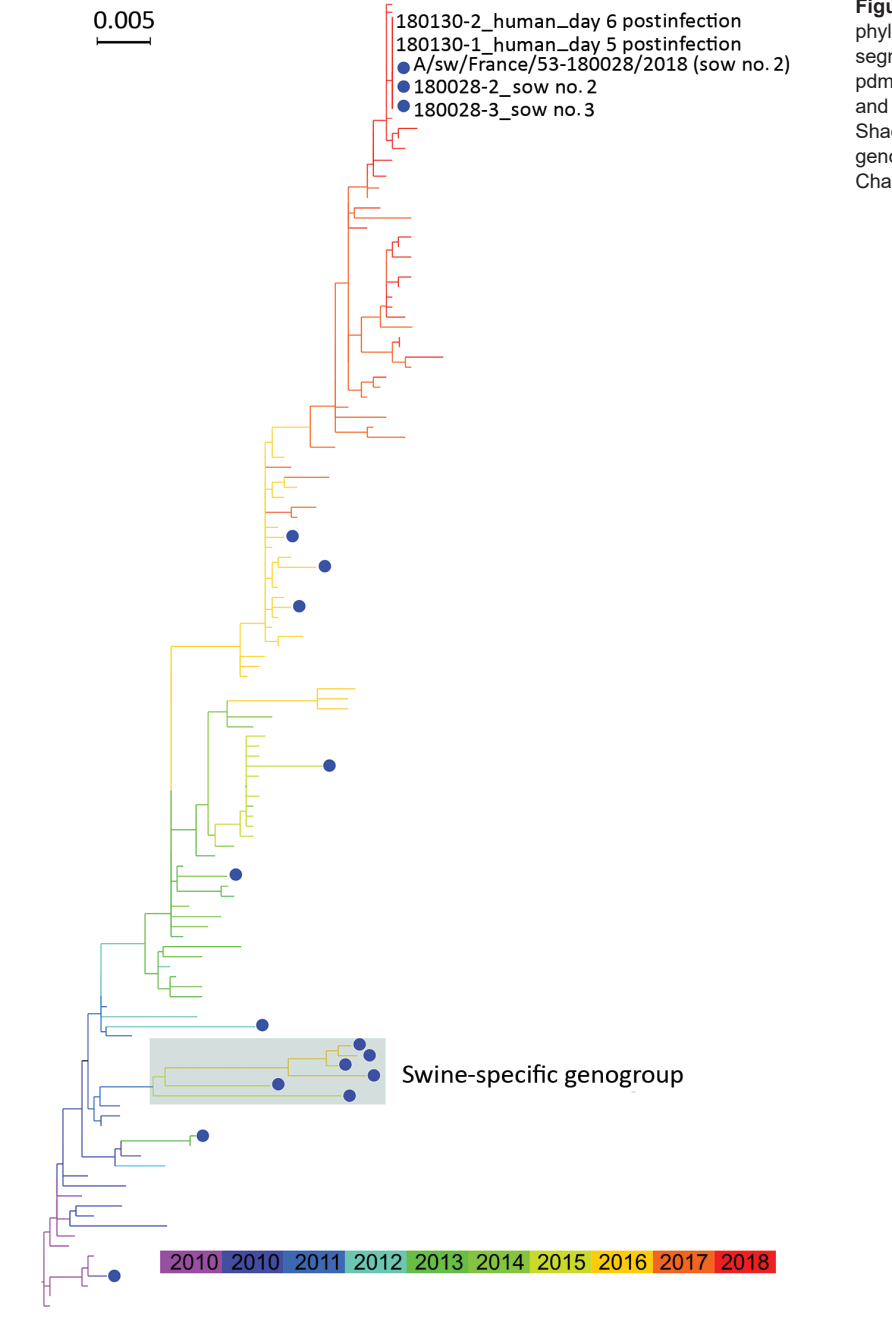
Maximum-likelihood phylogenetic tree of hemagglutinin segments from influenza A(H1N1)pdm09 isolates from swine (blue dots) and humans, France, 2009–2018. Shaded box indicates swine-specific genogroup previously described by Chastagner et al. (*6*).

The timing of events and results of analyses led to multiple hypotheses: that de novo human-to-swine pH1N1 virus transmission would have been responsible for the first infection in this herd, that swine-to-swine transmission within the herd would have then been responsible for additional animal infections, and that subsequent swine-to-human transmission would have been responsible for the infection in the veterinarian and probably also the one in the technician. Because gilts (i.e., <1-year-old female pigs) were not introduced into the herd during the weeks before the acute respiratory outbreak, the virus was most probably transmitted to sows by an infected person who entered the farm, probably an employee who, according to the farmer, displayed an ILI a few days before he spotted the first clinical signs in sows. This employee took a shower before entering the breeding area and put on dedicated clothes but did not wear a protective mask or gloves. Likewise, swine-to-human transmission was probably facilitated by the veterinarian and technician not wearing personal protective equipment when handling the sick sows. In either of these situations, transmission probably resulted from contact with respiratory secretions or inhalation of aerosols generated by shedding humans or animals or by contact with contaminated fomites (*10*).

Serologic investigations have previously suggested that occupational exposure to pigs is a risk factor for human infections (*11*), but events of bidirectional pH1N1 interspecies transmission have been rarely demonstrated. This report confirms pH1N1 virus can easily be transmitted between pigs and humans. Other swine IAVs were inherited completely or partially from human IAVs, but pH1N1 virus was suspected to be introduced to swine more frequently than other strains, potentially because the virus’s origin was probably swine (*5*). After such reverse zoonotic events, the strain might undergo evolutionary adaptation, as revealed by the previously identified swine-specific genogroup (*6*); in cases of further antigenic divergence, these strains could constitute novel threats for humans lacking cross-immunity. Moreover, because of co-infections with other swine IAVs, numerous reassortants bearing >1 pH1N1 genomic segment have been described worldwide, and some of these strains have become enzootic in pig populations (*3*,*5*). Such reassortants could also be an increased risk to the public health, as illustrated by many swine-to-human transmission events of swine IAVs containing the pH1N1 matrix gene during exhibition fairs in the United States (*12*). These transmissions have strongly increased the number of zoonotic infections reported since the last pandemic; only a few cases were reported before 2009 (*13*,*14*).

## Conclusions

The emergence of novel IAVs that threaten both human and swine health can be facilitated by the virus crossing species barriers (*4*,*15*). The concomitant pH1N1 virus infections we report emphasize the importance of implementing ad hoc biosecurity measures in pig farms to prevent interspecies virus transmission (*10*). Our evidence supports the One Health perspective of providing pig industry workers the annual seasonal influenza vaccination. This practice can minimize the risk for workers acquiring pH1N1 virus infections from pigs and for workers transmitting human IAVs to pigs.

Appendix Additional information on the bidirectional human–swine transmission of seasonal influenza A(H1N1)pdm09 virus in a pig herd in France, 2018.
