# Supplementary material for: Bidirectional Human–Swine Transmission of Seasonal Influenza A(H1N1)pdm09 Virus in Pig Herd, France, 2018
Source: Emerg Infect Dis. 2019 Oct;25(10):1940–3. doi: 10.3201/eid2510.190068 (PMC6759248; doi:10.3201/eid2510.190068)
Supplement: Appendix — Additional information on the bidirectional human–swine transmission of seasonal influenza A(H1N1)pdm09 virus in a pig herd in France, 2018. [file 19-0068-Techapp-s1.pdf]

Article DOI: https://doi.org/10.3201/eid2510.190068

Bidirectional Human–Swine Transmission of Seasonal Influenza A(H1N1)pdm09 Virus in Pig Herd, France, 2018

Appendix

Next Generation Sequencing (NGS) Methods Used to Obtain Whole Genome Consensus Sequences of Viruses Contained in Samples #180028–1, #180028–2, #180028–3, #180130–1, #180130–2, and A/Swine/France/53–180028/2018

Whole genome of strain A/Swine/France/53–180028/2018 was sequenced on the Agence Nationale de Sécurité Sanitaire de l'Alimentation, de l'Environnement et du Travail (ANSES) NGS platform (Ploufragan, France) on an Ion Proton Sequencer (Thermo Fischer Scientific Inc.) using the Ion Total RNA-Seq Kit v2 for preparation of cDNA libraries. The reads were cleaned up with Trimmomatic [version 0.36] and de novo assembled with SPAdes [version 3.10.0] and MIRA [version 4.0.2] (detailed protocol upon request). The de novo contigs were then submitted to MEGABLAST on a local nucleotide database and the best matches were selected for alignment with the Burrows-Wheeler Aligner (BWA) program [version 0.7.15-r1140]. Finally, all contigs were aligned and manually cleaned using ContigExpress (component of Vector NTI Advance 10.3.0) to produce a unique consensus sequence for each of the eight genomic segments.

In parallel, the viruses contained in samples #180028–1, #180028–2, and #180028–3 taken from sows and samples #180130–1 and #180130–2 taken from the veterinarian were sequenced by the National Reference Center for Respiratory Viruses (Paris, France). Amplification products from the eight viral segments were synthesized from the five RNA samples using universal primers essentially as described (*1*) and purified using the NucleoSpin Gel and PCR Cleanup kit (Macherey Nagel). NGS was performed by the Mutualized Platform for Microbiology (Paris, France) using the Nextera XT DNA Library Preparation kit (Illumina Inc.), the NextSeq 500 sequencing system (Illumina Inc.) and the CLC Genomics Workbench 11 software (QIAGEN) for pre-analysis.

For further analysis, reads were cleaned up using the Trimmomatic tool [version 0.36.4, ANSES Galaxy platform] and mapped on the A/California/07/2009 reference genome sequence using the BWA–MEM tool [version 0.7.12.1, ANSES Galaxy platform] to obtain consensus sequences.

References

1. Watson SJ, Welkers MR, Depledge DP, Coulter E, Breuer JM, de Jong MD, et al. Viral population analysis and minority-variant detection using short read next-generation sequencing. Philos Trans R Soc Lond B Biol Sci. 2013;368:20120205. PubMed https://doi.org/10.1098/rstb.2012.0205
